# Opinion Evolution in Divided Community

**DOI:** 10.3390/e24020185

**Published:** 2022-01-26

**Authors:** Tomasz Weron, Janusz Szwabiński

**Affiliations:** Hugo Steinhaus Center, Faculty of Pure and Applied Mathematics, Wrocław University of Science and Technology, 50-370 Wrocław, Poland; janusz.szwabinski@pwr.edu.pl

**Keywords:** opinion dynamics, social polarization, agent-based model, Monte-Carlo simulation

## Abstract

Our agent-based model of opinion dynamics concerns the current vast divisions in modern societies. It examines the process of social polarization, understood here as the partition of a community into two opposing groups with contradictory opinions. Our goal is to measure how mutual animosities between parties may lead to their radicalization. We apply a double-clique topology with both positive and negative ties to the model of binary opinions. Individuals are subject to social pressure; they conform to the opinions of their own clique (positive links) and oppose those from the other one (negative links). There is also a chance of acting independently, which alters the system’s behavior in various ways, depending on its magnitude. The results, obtained with both Monte-Carlo simulations and the mean-field approach, lead to two main conclusions: in such a system, there exists a critical quantity of negative relations that are needed for polarization to occur, and (rather surprisingly) independent actions actually support the process, unless their frequency is too high, in which case the system falls into total disorder.

## 1. Introduction

Polarization is a frequently used concept in social and political science as well as economics, but its definition may differ between domains. Within this paper, we will follow the one given by DiMaggio et al. and assume that polarization refers to a situation in which a group of people is divided into two opposing cliques with contrasting positions on a given issue [1]. This type of polarization is sometimes called bi-polarization [2] to distinguish it from the group polarization phenomenon, i.e., the tendency for a group to make more extreme decisions than the initial inclination of its members [3,4].

Recent observers point to a growing polarization of modern societies [5]. This seems to be a defining feature of many public domains and was identified in the World Economic Forum’s 2017 Global Risk Report as one of the top threats to the global order [6]. Consequently, it is gaining increasing attention from researchers working at the intersection of many fields, including social and political science, economics, mathematics and statistical physics.

High and increasing levels of polarization are attributed to a variety of sources, including the isolating effects of social media or news outlets focusing more on outraged rants than reasoned debates. Although significant progress has been observed in our understanding of polarization mechanisms in recent years, our knowledge remains sketchy and there is still a lot of room for improvement. Each new insight into polarization is important, because it is known to have a huge impact on societies. This leads to social tension and conflicts, and may result in the segregation of societies [1].

Interestingly, not all debates have the potential to polarize societies. From the observations, it follows that, in order to drive people to extreme and opposing opinions, the topic of a discussion has to be perceived as important and emotionally charged by all participants. That is why polarizing topics comprise controversial issues such as abortion rights, homosexuality, public funding for the arts, gun control, global warming, vaccination and, last but not least, politics [7,8,9,10,11,12].

Starting with Eli Pariser’s book [13], social media sites are increasingly blamed for intensifying (political) polarization. The artificial intelligence algorithms used by sites such as Facebook, Twitter or Google to profile the users create so-called “echo chambers” (or “filter bubbles”), which separate people from the information that disagrees with their viewpoints. The idea behind these algorithms was to let the people stay in their comfort zones. An unexpected side effect of this approach is an unconscious confirmation bias, because people are mainly confronted with information that reinforces their beliefs and opinions. The bias may contribute to overconfidence in personal beliefs and can maintain or strengthen them in the face of contrary evidence, which leads to polarization [5].

Several possible mechanisms leading to a stable bi-polar distribution of opinions within a simulation have been already proposed. There is, for instance, a series of papers showing that opinion homophily may support opinion plurality, including polarization [14,15,16,17]. This type of homophily is understood as a relationship between a similarity in peoples’ views and an increased likeliness of their interaction. This was usually implemented as a bounded confidence, i.e., threshold mechanisms that switch off influence in case the discrepancy in opinion is too big. Long-range ties (bridges between clusters) in a social network may also foster polarization if homophily and assimilation at the microlevel are combined with some negative influence, e.g., xenophobia [18,19]. From social balance theories, it follows that a mixture of positive and negative ties is needed for polarization to emerge and prevail [20,21,22]. In the argument-communication model, agents with a similar attitude mutually reinforce that attitude by the exchange of supportive arguments, which, in some circumstances, also leads to polarization [2]. Both the majority model [23] and the Ising model [24] in a segmented network only support the initial polarization in the presence of conforming relations if the density of connections between the segments remains low. Finally inflexibility, understood here as an internal opinion that encodes how many encounters with different-minded agents are needed for an agent to change its external opinion, has been shown to polarize a population in the sense that two opposing camps of increasingly inflexible supporters may emerge [25,26,27].

Recently, we proposed a simple polarization model based on the *q*-voter model with both conformity and anticonformity [21,22]. We considered the model of a double-clique social network, because it mimics the echo chambers that are observed on social media platforms as well as the interactions between their members. We found that if the number of inter-clique connections stays below a critical value, a consensus between two antagonistic cliques is possible. Thus, in light of these results, the artificial intelligence algorithms producing echo chambers on many platforms may have a positive impact in terms of polarization, because they reduce exposure to different opinions. In this paper, we are going to extend our model with independence to make the spectrum of possible responses to social influence more realistic from the social science perspective [28,29,30].

The paper is organized as follows. In the next section, we provide a detailed description of the models and methods used to analyze them. Then, we present the results. Finally, in Section 4, we discuss the results and draw some conclusions.

## 2. Models and Methods

### 2.1. Modelling Framework

The basic assumptions of our model have been already extensively discussed in Refs. [21,22]. Therefore, we start this section with only a short overview of its major premises:

- A binary opinion model with a single trait.
- *q*-voter model with conformity and anti-conformity as the general modeling framework.
- Double clique topology as the underlying social network.
- Conformity between agents within a clique and anti-conformity in the interactions between the cliques.

All of the above assumptions can be justified by recent findings in the opinion dynamics community. For instance, the analysis of many social networks revealed that the polarization of opinions within those networks may be correlated with their segmentation [31,32,33]. Hence, we assumed that the network is already modular and took the double-clique topology [34] as its model. Choosing a binary model with a single trait is rooted in the observation that, in many situations, people’s opinions may be interpreted as simple “yes/no” (i.e., binary) answers [35]. Moreover, social networks are often characterized by a semantic unicity, i.e., the opinions and interactions of network members are restricted to a single topic [36].

The *q*-voter model is one of the extensively studied models of binary opinions. Within the original formulation [37], the dynamics is given by the following update rule:

1. Pick a target agent at random.
2. Choose randomly *q* neighbors of the target (possibility of repetition).
3. If all the *q* neighbors are in the same state, the target changes its state accordingly.
4. Otherwise, the target changes its state with probability ϵ.

The unanimity rule embedded in the model is in line with a number of social experiments [38,39,40]. Please note that, in our studies, we only consider ϵ=0, following the setup in the previous papers [21,22].

Conformity, understood as the act of matching opinions to the group norm, is the only social force in the original *q*-voter model. However, it is relatively easy to extend it, with other possible responses, to social influences such as independence and/or anticonformity [30,41,42,43,44,45,46,47,48]. The first one is simply unwillingness to yield to group pressure and introduces noise to the system; the latter means a deliberate challenging of the group position. In Refs. [21,22], we used anticonformity to mimic negative ties between agents belonging to two opposite cliques, in agreement with the social balance theories [20]. It should be noted that the double-clique topology, with conformity inside a clique and anticonformity between the cliques, resembles, to some extent, the controversial echo chambers generated by social platforms [13].

### 2.2. Independence of Agents

In Refs. [21,22], we have shown, both theoretically and by means of Monte Carlo simulations, that a system consisting of two connected antagonistic cliques undergoes a phase transition as the number of cross-links between the cliques changes. Below the critical point (i.e., loosely connected cliques), the intra-clique conformity takes over and consensus in the entire system is possible as an asymptotic state. Above the critical point, the system ends up in a polarized state, with the cliques having opposite opinions and a local consensus between them. This was a surprising result, because it actually defies the criticism of echo chambers that was started by Pariser [13]. Since the algorithms generating the echo chambers reduce the exposure time to different-minded people, in light of our findings, they should lower the polarization level between antagonistic groups, instead of enhancing it.

However, one of the drawbacks of the model presented in Refs. [21,22] was the lack of independence in the behavior of agents. This concept has been already considered in a series of models [30,41,49,50,51,52]. It actually implies the failure of an attempted social influence, because an independent individual makes decisions independently of the group norm. From the perspective of social science, it falls (together with anticonformity) into the category of non-conformal behaviors [28,29,53]. From a physical point of view, it plays the role of social temperature that induces an order–disorder transition [41,54,55]. Thus, it would be interesting to check how the introduction of independence into our model will change the behavior of the entire system, and if our findings still hold in the extended version of the model.

**Figure 1:**
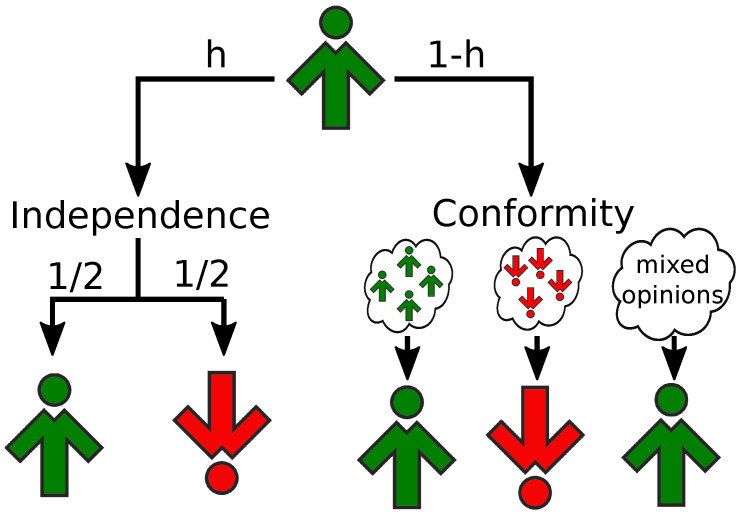
Schematic representation of the opinion update of a single agent that was initially in the up state. With probability *h*, the agent acts independently and changes opinion randomly. With complementary probability 1−p, the agent is subject to social influence.

We will introduce the independence to the model in a situation-oriented manner [44,46]. In a given time step, a target of influence will behave independently with probability *h* or will become a conformist with probability 1−h (Figure 1). Thus, an additional control parameter *h* will be used to simulate the impact of the situational factors on the behavior of agents. Within this approach, every agent may change his behavior from step to step, and sometimes act independently, and sometimes like a conformist (see Section 2.4 for detailed, updated rules of the model).

### 2.3. Quenched and Annealed Disorder Models

In Ref. [22], two versions of the model were considered. In the quenched disorder one, two cliques of size *N* are connected with $L\times N^{2}$ cross-links. The parameter *L* is simply the fraction of the existing cross-links; $N^{2}$ is their maximum number. Once the links between cliques are chosen randomly, they remain fixed—the resulting network does not change in time during the evolution of the system.

Instead of working with the fixed-cross links, in the annealed version of the model, we assume that every agent from one clique is connected with probability *p* with an agent from the other clique, and with probability 1−p, with an agent from its own clique. Technically, this approximation is nothing but an average of the quenched disorder model over different cross-link configurations in the network.

Given the fraction of existing cross-links *L*, the probability *p* of choosing one cross-link out of all possible connections between agents in the double-clique network is given by

(1) $$p=\frac{LN^{2}}{LN^{2}+2\frac{N(N-1)}{2}}≃\frac{L}{L+1}.$$

If the number of cross-links is smaller than their maximum number, the agents in the quenched disorder model differ from each other, because some of them may have no connections to the other clique, while some others have multiple ones. While it can be handled with ease within a computer simulation, this feature usually constitutes a challenge for mathematical modeling due to the necessity of performing a quenched average over the disorder [56]. The annealed model is easier, in the sense that it allows for mathematical treatment.

### 2.4. Updating Rules of the Models

To recap, we consider a set of 2N agents, each of whom may be in one of two possible states, reflecting an opinion on some given issue: $S_{i}=-1$ or $S_{i}=1$ for i=1,2,…,2N. We put the agents into a double-clique network, which consists of two complete graphs of *N* nodes connected with $L\times N^{2}$ cross-links.

**Figure 2:**
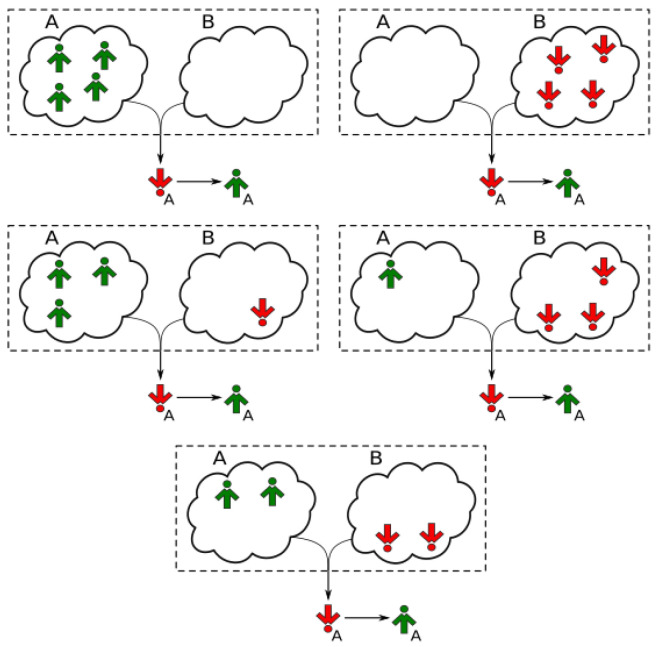
All possible choices of the influence group in the model with q=4 that lead to an opinion flip by a target from clique *A* that was initially in state S=−1. The influence group may contain members from both cliques. Due to the presence of both positive and negative ties, the concept of unanimity from the original *q*-voter model has to be extended to signals, which are then received by the target of influence. A signal is the state of a member when coming from target’s clique, or its inverted state otherwise. The target changes its opinion only if all members of the influence group emit the same signal.

We assume that the social response of agents depends on their group identity. Thus, an agent will strive for agreement within his/her own clique (conformity) and simultaneously challenge the opinions of individuals from the other clique (anticonformity). As in Ref. [22], we introduce the notion of signals to the *q*-voter model and slightly alter the concept of unanimity of the influence group in order to account for the fact that an agent may act as both a conformist and anticonformist at the same time. A signal is just the state of the neighbor when coming from the target’s clique, or its inverted state otherwise. The target of influence only changes its opinion if all members of the influence group emit the same signal (Figure 2).

We will use Monte Carlo simulations with a random sequential updating scheme as the main tool to analyze the models. Each Monte Carlo step in a simulation consists of 2×N elementary events, each of which may be divided into the following substeps with $\Delta t=\frac{1}{2N}$:

1. Pick a target agent at random (uniformly from 2N nodes).
2. Draw a random number form a uniform distribution, r∼U(0,1).
3. If r<h (i.e., with probability *h*), the agent is independent:
  (a) Change its state with probability 1/2. To this end, draw a random number *f* from a uniform distribution, f∼U(0,1):
    - if f<1/2, change the state of the target, i.e., $S_{i}\left(t+\Delta t\right)=-S_{i}\left(t\right)$,
    - otherwise, do nothing.
  (b) Go to step 1.
4. If r>h (i.e., with probability 1−h), the agent is subject to social influence:
  (a) Randomly choose a group of *q* distinct neighbors of the target node:
    Quenched model simply look at the actual neighbors of the target (sampling with replacement).
    Annealed model first decide to which clique every member of the influence group will belong (with probability 1−p to the target’s clique, with *p* to the other one), then randomly choose the member from the appropriate clique.
  (b) Convert the states of the group members to signals. Assume that the signals of the neighbors from target’s clique are equal to their states. Invert the states when from the other clique.
  (c) Calculate the total signal of the influence group by summing up the individual signals of its members.
  (d) If the total signal is equal to ±q (i.e., all group members emit the same signal), the target changes its opinion accordingly (see Figure 2). Otherwise, nothing happens.
5. Go to step 1.

### 2.5. Quantities of Interest

The macroscopic state of an opinion dynamics model is usually described by either the concentration of agents in state +1 or the average opinion (i.e., magnetization in physical systems). Noting that the total number of agents in our model is 2N, we obtain the following formula for the concentration:

(2) $$c\left(t\right)=\frac{N^{↑}\left(t\right)}{2N}.$$

Here, $N^{↑}\left(t\right)$ stands for the number of agents in state +1. Similarly, the average opinion is given by

(3) $$m\left(t\right)=\frac{1}{2N}\sum_{i=1}^{2N}S_{i}=\frac{N^{↑}\left(t\right)-N^{↓}\left(t\right)}{2N},$$

where $N^{↓}\left(t\right)$ denotes the number of agents in state −1. Both quantities may be used interchangeably, because

(4) m(t)=2c(t)−1.

Knowing the concentration of the entire system may be not enough to describe it uniquely in case of the double-clique topology. For instance, the value c(t)=1/2 may correspond to no ordering in the system (i.e., a perfect mixture of +1 and −1 states in both cliques) or to polarization (all agents in state +1 in one clique and in state −1 in the other). This is why it would be more insightful to calculate the above quantities for single cliques rather than for the entire system,

(5) $$\begin{array}{ccc} c_{X}\left(t\right) & = & \frac{N_{X}^{↑}\left(t\right)}{N},\;\;X=A,B,\\ m_{X}\left(t\right) & = & \frac{1}{N}\sum_{i=1}^{N}S_{X,i}=\frac{N_{X}^{↑}\left(t\right)-N_{X}^{↓}\left(t\right)}{2}. \end{array}$$

The interpretation of their values is summarized in Table 1. Moreover, from the above definition, it follows that $c_{X}$ may be interpreted as the probability of finding an agent in state 1 within the clique *X*.

It is also interesting to look at the product $m_{A}\left(t\right)m_{B}\left(t\right)$ of the clique magnetizations, as it immediately indicates a consensus (the value of the product equal to 1) and polarization (−1) for the entire system.

### 2.6. Transition Probabilities and Dynamical System

The random sequential updating scheme in our model means that, in each time step Δt=1/2N, only one agent can change its opinion. Three scenarios are possible: (1) the total amount of agents in state +1 in a clique may increase by 1 within this step, (2) the total amount may decrease by 1 or (3) it may remain unchanged.

Let us have a look at the first of the above scenarios. The number of agents in state +1 in one clique—say A—can increase by 1 only if:

1. a target from clique *A* is chosen (probability 1/2),
2. the target is in state −1 (probability $1-c_{A}$),
3. it flips due to independence (probability h/2) or follows an influence group emitting signal +q.

Thus, the transition probability for such an event will be given by

(6) $$\begin{array}{c} \mathrm{Pr}\left\{N_{A}^{↑}\left(t+\Delta t\right)=N_{A}^{↑}\left(t\right)+1\right\}=\\ \;\;\frac{1}{2}\left(1-c_{A}\left(t\right)\right)\left(\frac{1}{2}h+\left(1-h\right){\left[\left(1-p\right)c_{A}\left(t\right)+p\left(1-c_{B}\left(t\right)\right)\right]}^{q}\right). \end{array}$$

One can easily check that the term of the form ${\left(u+v\right)}^{q}$ in the above equation is the generating function for the probabilities of those compositions of *q* members of an influence group that can cause an opinion-switch event (see Figure 2 for more details). Similarly, the number of agents in state +1 in clique *A* decreases by 1 if:

1. A target from clique *A* is chosen (probability 1/2).
2. The target is in state +1 (probability $c_{A}$).
3. It flips due to independence (probability h/2) or follows an influence group emitting signal −q.

These conditions lead to the following transition probability:

(7) $$\begin{array}{c} \mathrm{Pr}\left\{N_{A}^{↑}\left(t+\Delta t\right)=N_{A}^{↑}\left(t\right)-1\right\}=\\ \;\;\frac{1}{2}c_{A}\left(t\right)\left(\frac{1}{2}h+\left(1-h\right){\left[\left(1-p\right)\left(1-c_{A}\left(t\right)\right)+pc_{B}\left(t\right)\right]}^{q}\right). \end{array}$$

It is also possible that the number of agents in state +1 remains unchanged in an elementary time step. The probability of this event is 1 minus the above probabilities of changes:

(8) $$\begin{array}{c} \mathrm{Pr}\left\{N_{A}^{↑}\left(t+\Delta t\right)=N_{A}^{↑}\left(t\right)\right\}=\\ \;\;1-\mathrm{Pr}\left\{N_{A}^{↑}\left(t+\Delta t\right)=N_{A}^{↑}\left(t\right)+1\right\}-\mathrm{Pr}\left\{N_{A}^{↑}\left(t+\Delta t\right)=N_{A}^{↑}\left(t\right)-1\right\}. \end{array}$$

Analogous considerations can be conducted for clique *B*.

Given the states of the cliques at time *t* and the above transition probabilities, the expectations for the numbers of agents in state +1 at time t+Δt may be written as

(9) $$\begin{array}{ccc} E\left(N_{A}^{↑}\left(t+\Delta t\right)\right)=N_{A}^{↑}\left(t\right) & + & \frac{1}{2}\left(1-c_{A}\left(t\right)\right)\left(\frac{1}{2}h+\bar{h}{\left[\bar{p}c_{A}\left(t\right)+p\left(1-c_{B}\left(t\right)\right)\right]}^{q}\right)\\ & - & \frac{1}{2}c_{A}\left(t\right)\left(\frac{1}{2}h+\bar{h}{\left[\bar{p}\left(1-c_{A}\left(t\right)\right)+pc_{B}\left(t\right)\right]}^{q}\right),\\ E\left(N_{B}^{↑}\left(t+\Delta t\right)\right)=N_{B}^{↑}\left(t\right) & + & \frac{1}{2}\left(1-c_{B}\left(t\right)\right)\left(\frac{1}{2}h+\bar{h}{\left[\bar{p}c_{B}\left(t\right)+p\left(1-c_{A}\left(t\right)\right)\right]}^{q}\right)\\ & - & \frac{1}{2}c_{B}\left(t\right)\left(\frac{1}{2}h+\bar{h}{\left[\bar{p}\left(1-c_{B}\left(t\right)\right)+pc_{A}\left(t\right)\right]}^{q}\right), \end{array}$$

where the abbreviations $\bar{p}=1-p$ and $\bar{h}=1-h$ have been introduced for the sake of readibility.

Under the very likely assumption that the random variables $c_{A,B}=\frac{N_{A,B}^{↑}}{N}$ localize in the limit N→∞, after the division of both sides of the equations by *N*, we obtain

(10) $$\begin{array}{ccc} \frac{c_{A}\left(t+\Delta t\right)-c_{A}\left(t\right)}{\Delta t} & = & \left(1-c_{A}\left(t\right)\right)\left(\frac{1}{2}h+\bar{h}{\left[\bar{p}c_{A}\left(t\right)+p\left(1-c_{B}\left(t\right)\right)\right]}^{q}\right)\\ & & -c_{A}\left(t\right)\left(\frac{1}{2}h+\bar{h}{\left[\bar{p}\left(1-c_{A}\left(t\right)\right)+pc_{B}\left(t\right)\right]}^{q}\right),\\ \frac{c_{B}\left(t+\Delta t\right)-c_{B}\left(t\right)}{\Delta t} & = & \left(1-c_{B}\left(t\right)\right)\left(\frac{1}{2}h+\bar{h}{\left[\bar{p}c_{B}\left(t\right)+p\left(1-c_{A}\left(t\right)\right)\right]}^{q}\right)\\ & & -c_{B}\left(t\right)\left(\frac{1}{2}h+\bar{h}{\left[\bar{p}\left(1-c_{B}\left(t\right)\right)+pc_{A}\left(t\right)\right]}^{q}\right). \end{array}$$

In the limit N→∞, i.e., $\Delta t=\frac{1}{2N}\to 0$, we arrive at the dynamical system representing the annealed model:

(11) $$\begin{array}{ccc} \frac{dx}{dt} & = & \left(1-x\right)\left(\frac{1}{2}h+\bar{h}{\left[\bar{p}x+p\left(1-y\right)\right]}^{q}\right)-x\left(\frac{1}{2}h+\bar{h}{\left[\bar{p}\left(1-x\right)+py\right]}^{q}\right),\\ \frac{dy}{dt} & = & \left(1-y\right)\left(\frac{1}{2}h+\bar{h}{\left[\bar{p}y+p\left(1-x\right)\right]}^{q}\right)-y\left(\frac{1}{2}h+\bar{h}{\left[\bar{p}\left(1-y\right)+px\right]}^{q}\right), \end{array}$$

where *x* and *y* are the limiting values of concentrations $c_{A}$ and $c_{B}$, respectively.

## 3. Results

We will assume that the number of agents in every clique in the quenched model is N=100. Although the size of the system may seem very small, it was already shown in Refs. [21,22] that increasing the size does not qualitatively change the outcome of the simulations, but it takes substantially longer to finish them.

In our analysis, we considered influence groups of sizes ranging from 2 to 6, with the upper bound motivated by the conformity experiments by Asch [40]. Qualitatively, the results were independent of the actual value of *q*. Thus, we decided to present the results for q=4, a value often used in the analysis of the *q*-voter model and its extensions.

If not stated otherwise, the results of the simulations were averaged over 1000 independent runs. In most cases, the asymtotic state was reached quickly, in less than 100 Monte Carlo steps. We used our own codes written in C++, Python and Matlab.

As for the initial condition, we used the total positive consensus, i.e., all agents in the state +1. As already pointed out in Ref. [21], this choice may be treated as a result of the following scenario. Two cliques with a natural tendency to disagree with each other first evolve independently. They get in touch by chance and establish some cross-links to the other group after they both reach consensus on a given issue.

When comparing the two models, quenched and annealed, we present the results with respect to the fraction of the existing cross-links, *L*, instead of *p*, using the relationship from Equation (1).

### 3.1. Direction Fields and Stationary Points

**Figure 3:**
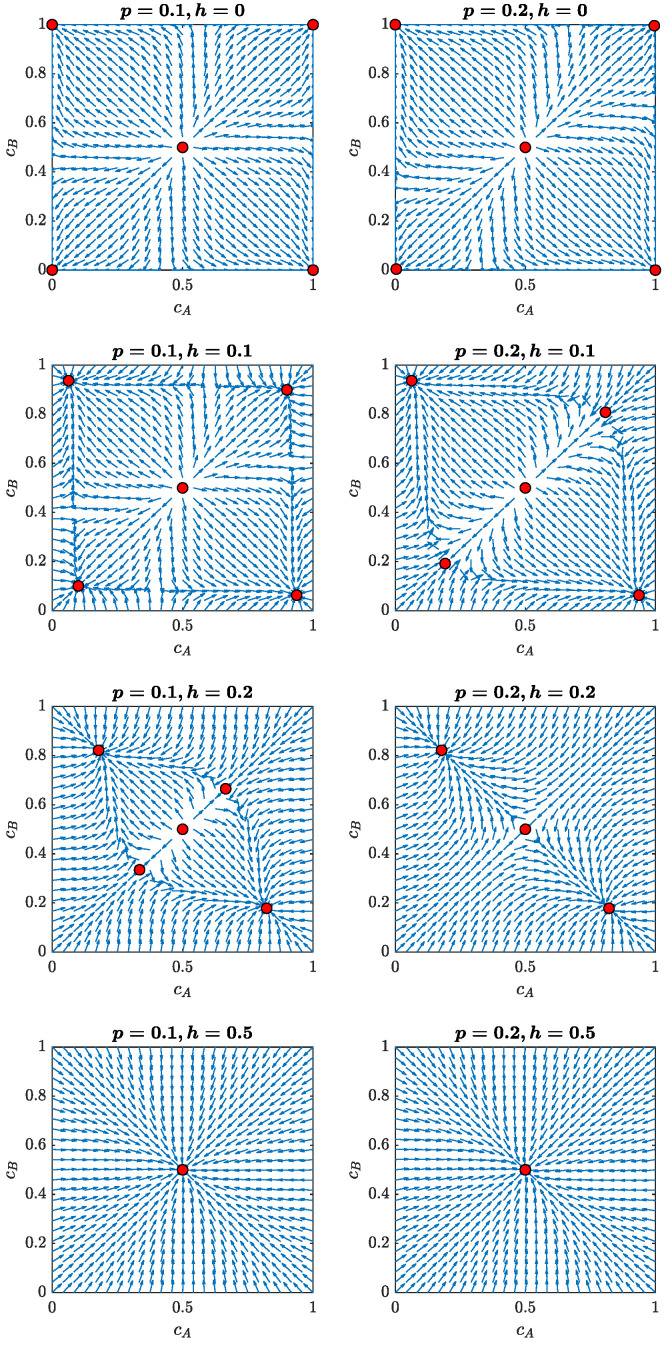
The annealed model: direction fields of the model described by Equation (11) with fixed points marked with circles for different values of independence *h* and two values of parameter *p*, 0.1 (**left** column) and 0.2 (**right** column).

The set of Equations (11) is too cumbersome to solve analytically. However, we still can generate direction fields for the set to graphically trace out solution curves for various initial values [57]. Results for different independence levels *h* and two different probabilities of an inter-clique connection *p* are shown in Figure 3: the left column contains the plots for p=0.1; the right one corresponds to p=0.2. The values of *h* are equal to 0.0, 0.1, 0.2 and 0.5 (from top to bottom). Note that the case h=0 is nothing but our original model with no independence, which was extensively studied in Ref. [22].

From the flows in the state plane, it follows that for p=0.1 and h=0, there are five stationary points (already marked with dots in the plots). Two attractors, $P_{1}=\left(0,1\right)$ and $P_{2}=\left(1,0\right)$, correspond to a polarized state of the system, i.e., all agents in one clique are in state +1 and in the others are in state −1. There are two other symmetric attractors, $C_{1}$ and $C_{2}$, which are very close to the coordinates (0,0) and (1,1). Thus, the state of (almost) complete consensus is possible in the system as well. The remaining point *R* is a repeller, because the system tends to evolve away from it.

To find the exact coordinates of the stationary points, we set $x^{\prime }$ and $y^{\prime }$ as equal to zero in Equation (11) and solve the resulting set of equations with respect to *x* and *y*,

(12) $$\begin{array}{ccc} 0 & = & \left(1-x\right)\left(\frac{1}{2}h+\bar{h}{\left[\bar{p}x+p\left(1-y\right)\right]}^{q}\right)-x\left(\frac{1}{2}h+\bar{h}{\left[\bar{p}\left(1-x\right)+py\right]}^{q}\right),\\ 0 & = & \left(1-y\right)\left(\frac{1}{2}h+\bar{h}{\left[\bar{p}y+p\left(1-x\right)\right]}^{q}\right)-y\left(\frac{1}{2}h+\bar{h}{\left[\bar{p}\left(1-y\right)+px\right]}^{q}\right). \end{array}$$

For p=0.1 and h=0.0, we obtain:

(13) $$\begin{array}{ccc} & & P_{1}=\left(0,1\right),\;\;\;\;P_{2}=\left(1,0\right),\\ & & C_{1}=\left(0.00015,0.00015\right),\;\;\;\;C_{2}=\left(0.99985,0.99985\right),\\ & & R=(0.5,0.5). \end{array}$$

Introducing a small level of independence (h=0.1 and 0.2) into the model does not change the classification of the stationary points for p=0.1. However, they are now shifted towards the center of the state plane, meaning that complete polarization and (almost) complete consensus have changed to partial ones. Although these states are still characterized by a majority of agents sharing the same opinion, due to the fluctuations induced by independence there is now always a minority with the opposite opinion. At a high independence level (h=0.5), the point R=(0.5,0.5) becomes an attractor and the other stationary points disappear.

The situation for p=0.2 is similar, but the effects induced by the independence *h* are stronger. This is why we explicitly see a state with only three fixed points at h=0.2 (the same state for p=0.1 would require 0.2<h<0.3 and is not shown in Figure 3). We can see that, in this case, the consensus attractors $C_{1}$ and $C_{2}$ have already disappeared. The polarization ones are still there, but are closer to the center of the plane. The repeller R=(0.5,0.5) becomes hyperbolic. With further increases in *h*, the polarization attractors will disappear as well and point *R* will become an attractor (see case h=0.5).

Compared to the model without independence [21,22], we observe an additional dynamical phase transition in the system—for high enough independence levels, it enters the disordered phase with the vanishing magnetization in every clique, as the asymptotic state.

### 3.2. Time Evolution of the System

**Figure 4:**
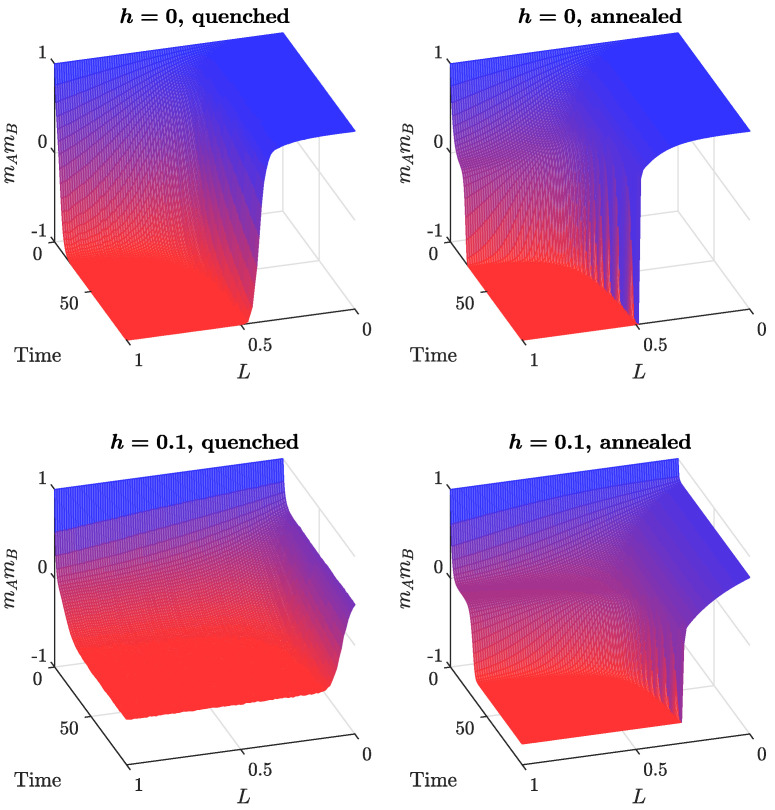
Comparison between the quenched (**left** column) and annealed (**right** column) models: product of magnetizations $m_{A}m_{B}$ as a function of time and *L*, for two different independence levels, h=0,0.1.

**Figure 5:**
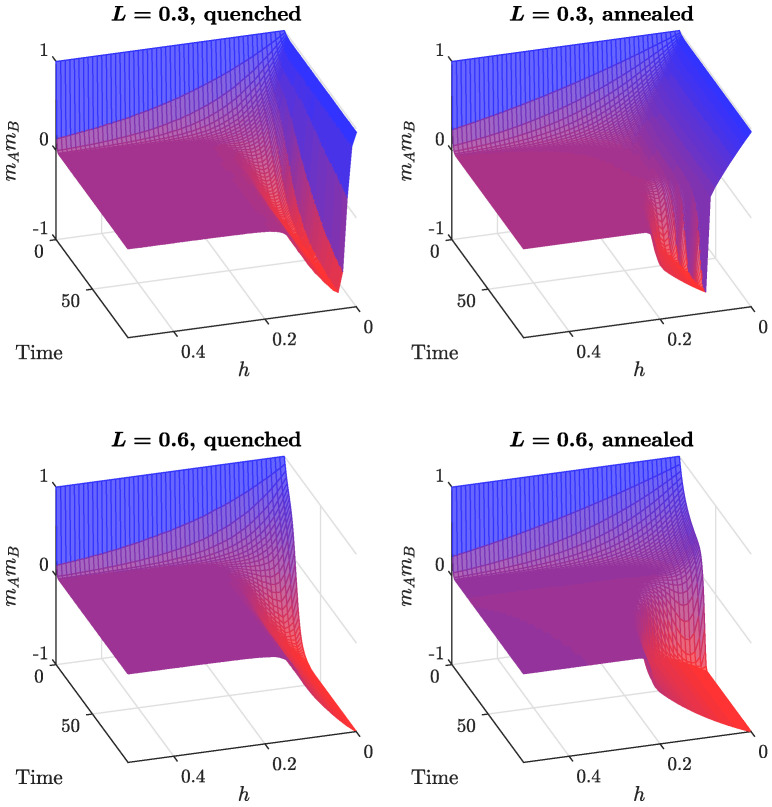
Comparison between the quenched (**left** column) and annealed (**right** column) models: product of magnetizations $m_{A}m_{B}$ as a function of time and *h*, for two different fractions of cross-links, L=0.3,0.6.

The asymptotic dynamical system for the annealed model, given by Equation (11), was solved numerically. Results for different values of *h*, as a function of time and *L*, are shown in Figure 4 (right column). As was already concluded from the direction fields (Figure 3), in the absence of independence (top right plot in Figure 4), a consensus is observed in both cliques for a low number of cross-links. More connections between the cliques drive the system towards a polarized state. The picture is different for a low level of independence in the model (bottom right plot in Figure 4). We still observe a consensus if the cliques are poorly connected. However, polarization sets in at a much lower number of cross-links. Moreover, both the consensus and polarization are partial, because, due to independence, there is always a group of agents that do not go along with the majority. Increasing the independence level destroys the ordering in the system and the model ends up in an asymptotic state with no polarization (see Figure 5, right column). This last result is independent of the number of cross-links between the cliques.

Monte Carlo simulations of the quenched version of the model produce a similar output (see Figure 4 and Figure 5). However, the critical value of *L* for the dynamical consensus–polarization phase transition is smaller for the quenched model, in agreement with our previous findings for models without independence [21,22]. Moreover, in the quenched model, the inclusion of independence has a much greater impact (see Figure 4, bottom part).

### 3.3. Impact of Independence on the System

All results up to this point suggest that there are three effects resulting from the introduction of independence into the models: (1) final concentrations of agents sharing the same opinion are diminished, (2) the critical values of *L* at the consensus–polarization transition are smaller and (3) an additional dynamical phase transition from the polarized state to a disordered one occurs in the system.

To elaborate on those findings, let us have a look at Figure 4. The case h=0 (no independence) corresponds to the original models from Refs. [21,22]. We see that, for values smaller than a critical value, $L^{*}$, both cliques end up reaching a consensus. In other words, in this regime, the intra-clique conformity wins with the inter-clique anti-conformity, and both communities are able to maintain their initial consensus, at least partially. Larger values of *L* are needed for the negative ties to take over and push the system into a polarized state.

The impact of independence is two-fold. First, the final magnetizations have been pushed away from the values ±1 even in the case L=0, i.e., the total consensus changed to a partial one. Since this corresponds to the weakening of the force exerted by conformity, one would expect that, in this case, fewer cross-links are needed between the cliques to polarize the system. Indeed, the critical value of *L* decreases with an increasing independence *h*.

It should be noted that, for each value of *h*, there is a difference in the critical values $L^{*}$ between the quenched and annealed models. This is mainly a consequence of different system sizes—while Equation (11), defining the annealed model, was derived for an infinite system, we used only 200 agents in the simulations of the quenched one. It has been shown in Ref. [22] that the discrepancy between the models decreases with the increase in the size of the simulated system. We expect the models to converge for N→∞, despite the subtle changes in their dynamics.

**Figure 6:**
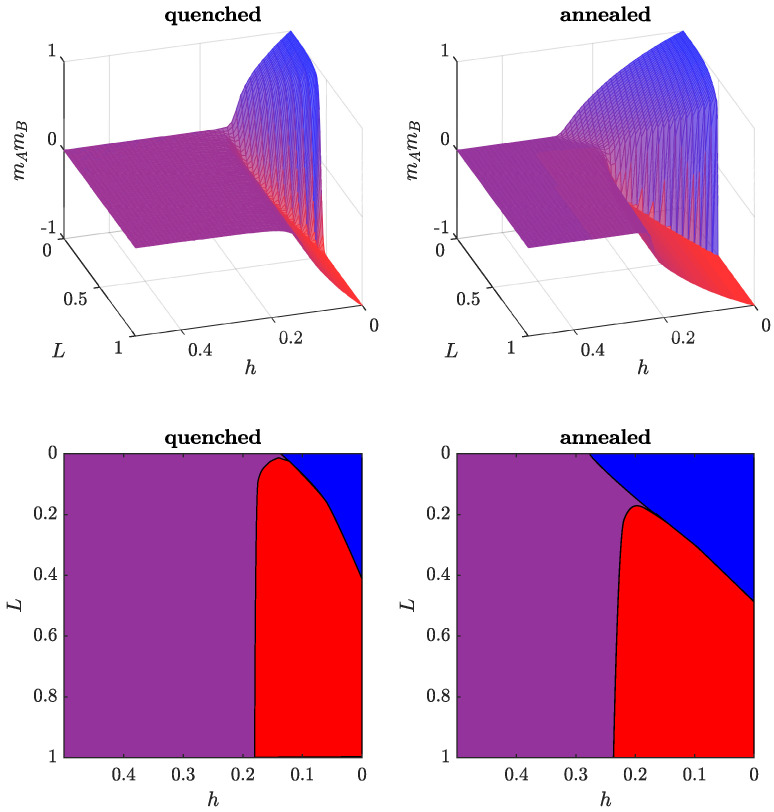
Comparison between the quenched (**left**) and annealed (**right**) models: final product of magnetizations $m_{A}m_{B}$ (**top**) and its projection on the (h,L) plane (**bottom**). The blue, red and purple colors correspond to consensus, polarization and disorder, respectively. In both models, we can observe that the critical value $L^{*}$ decreases with an increase in *h*, while *L* has only a marginal impact on $h^{*}$.

To complete this picture, let us investigate how the product of magnetization changes with both fraction of cross-links *L* and independence *h* (Figure 6). At L=0 (no connection between the cliques), independence continuously destroys the ordering in both communities. Finally, above a critical value $h^{*}$, the system enters the disordered phase with no magnetization in the cliques. For $L<L^{*}$ and small values of *h*, the system maintains the partial consensus, then we observe a transition to the polarized state. The magnetizations in the now-antagonistic cliques are diminishing with further increases of *h*. Finally, the system reaches the disordered phase. At $L>L^{*}$, the system is already polarized, even for h=0. Increasing *h* introduces disorder into the cliques. Again, there is no ordering above the critical value of *h*.

As already discussed earlier in this section, there are some differences in absolute values between the annealed and quenched models, but the picture for the annealed case is qualitatively the same.

It is worth noting that the critical value of *h* for the polarization–disorder transition depends little on *L* (with a more noticeable effect in the annealed model). At the same time, the critical value of *L* for the consensus–polarization transition decreases with an increasing *h*, unless the value *h* is too high and disorder becomes the only possible state (see Figure 6).

## 4. Discussion

The most important message from our previous study was that the consensus between two antagonistic communities is only possible if they are loosely connected with each other and the initial state of the system belongs to the basin of attraction of the symmetric fixed-points of the model [21,22]. The more interactions there are between those communities, the less probable it is that the entire system will share the same opinion. Instead, anticonformity takes over and pushes the system towards polarization. Those results were unexpected in the sense that they, for instance, support the idea of the often-criticized filter bubbles in social media [5,13]. Since those bubbles separate users from information that disagrees with their viewpoints, they may help to weaken the problem with polarization. However, the models that we considered were very simple. For instance, they lacked some typical answers to social influence [29].

In order to make the models more realistic, in this work, we added independence as a response to social influence. From our results, it follows that this additional manifestation of social interactions impacts the system dynamics in at least two ways. Small independence levels help anticonformity to take over and polarize the society. More technically speaking, they lower the critical ratio of cross-links between cliques, which are needed to arrive at a polarized state. High independence levels destroy any ordering in the system. Consequently, the opinions of agents are perfectly mixed across the cliques, and neither consensus nor polarization are observed. Instead, a third phase–a disordered state–appears.

In sum, in the presented setting, low (but present) independence levels seem to enhance the polarization of the system. Thus, they counteract the effects of the filter bubbles, which, at least within our models, foster consensus across the cliques. At high levels, all manifestations of the interplay between conformity and anticonformity are suppressed by the noise induced due to independence.

It is worth mentioning that qualitatively similar results (but with a less detailed stability analysis) were obtained earlier within the majority model [23] and the Ising model [24] on a double-clique topology, with conformity as the only response to social influence. Although, in those models, the initial polarized state was found to become unstable with the increasing number of connections between the cliques (the consensus one in our case), the dynamics of those models turned out to be very similar to the behavior presented in this paper. Unique to our model is a more realistic response of agents to social influence. In fact, we took all types of responses into account, according to the diamond model by Nail et al. [28,29,53]. Hence, one may expect that what has been observed is more a general pattern of social behavior than an artifact of a particular choice of the modeling framework.

## Figures and Tables

**Table 1:** Interpretation of different values of the concentration $c_{X}\left(t\right)$ and the average opinion $m_{X}\left(t\right)$ within a single clique *X* (see Equation (5) for definitions).

Meaning	$c_{X}\left(t\right)$	$m_{X}\left(t\right)$
Positive consensus (all agents in clique *X* in state +1)	$c_{X}=1$	$m_{X}=1$
Partial positive consensus (majority of agents in clique *X* in state +1)	$1/2<c_{X}<1$	$0<m_{X}<1$
No ordering in clique *X*	$c_{X}=1/2$	$m_{X}=0$
Partial negative consensus (majority of agents in clique *X* in state −1)	$0<c_{X}<1/2$	$-1<m_{X}<0$
Negative consensus (all agents in clique *X* in state −1)	$c_{X}=0$	$m_{X}=-1$

## Data Availability

Not applicable.
